# Brain Connectivity and Mental Illness

**DOI:** 10.3389/fpsyt.2012.00072

**Published:** 2012-07-27

**Authors:** Alex Fornito, Ben J. Harrison

**Affiliations:** ^1^Centre for Neural Engineering, The University of MelbourneParkville, VIC, Australia; ^2^NICTA Victorian Research Laboratory, The University of MelbourneParkville, VIC, Australia; ^3^Department of Psychiatry, Melbourne Neuropsychiatry Centre, The University of Melbourne and Melbourne HealthParkville, VIC, Australia

Despite nearly three decades of neuroimaging research in psychiatry, no imaging study to date has identified a single site of pathology in the brain that represents a causal factor in the emergence of any of the major mental illnesses described in current nosological systems. Rather, the available evidence indicates that these disorders are associated with subtle abnormalities distributed throughout the brain (e.g., Fornito et al., 2009, 2012; Bora et al., 2010, 2012), implying that they arise from disordered interactions between connected neural systems rather than damage to any individual brain region. Accordingly, current hopes for the development of more targeted interventions in psychiatry are being placed on research attempting to map the molecular determinants and clinical correlates of neural circuit abnormalities in mental disorders (Insel and Scolnick, 2006; Meyer-Lindenberg, 2010). The impact of this thinking on the field is evident in the emergence of new scientific journals devoted to the study of brain connectivity^1^; increasingly popular workshops devoted to the topic^2^; recent large-scale collaborative initiatives such as the Human Connectome Project^3^^,^^4^; freely available software packages dedicated to connectivity analyses^5^^,^^6^; and the establishment data-sharing initiatives such as the 1000 connectomes project^7^. These developments offer the potential to greatly enhance our understanding of brain connectivity abnormalities in psychiatric disorders.

This Special Topic of *Frontiers in Psychiatry* presents work illustrating the application of connectomic techniques to study brain network changes in mental disorders. It brings together researchers working to understand brain network abnormalities in disorders as diverse as schizophrenia, attention-deficit hyperactivity disorder (ADHD), major depression, bipolar disorder, post-traumatic stress disorder (PTSD), and Alzheimer's disease, and showcases the diverse range of methods used to interrogate these brain changes.

The article by Fornito and Bullmore (2012) overviews these techniques and reviews their application to the study of genetic influences on brain networks. They conclude that the available literature, albeit preliminary, indicates that risk genes show greater penetrance at the level of distributed brain networks rather than individual regions, and connectomic measures may thus provide more sensitive intermediate phenotypes for psychiatric disorders.

The remaining articles illustrate the application of specific techniques to study different disorders. Independent component analysis (ICA), a popular data-driven technique for decomposing fMRI data into distinct components with characteristic anatomy and temporal dynamics (Calhoun et al., 2004), is applied by Calhoun et al. (2011) to demonstrate phenotypic continuities and discontinuities in the brain functional networks of patients with schizophrenia and bipolar disorder performing an auditory oddball task.

Seed-based techniques are featured in the work of Mills et al. (2012), Mennes et al. (2011), Rabinak et al. (2011), and Davey et al. (2012). The first two articles use multiple seeds to characterize how individual differences in functional connectivity relate to cognitive performance on tests of executive function in children with ADHD and typically developing controls. This work offers welcome new evidence supporting the behavioral relevance of resting-state measures for psychiatric disorders, which is critical for validating any case-control differences observed under such experimental conditions (Fornito and Bullmore, 2010). In war veterans with combat-related PTSD, Rabinak et al. (2011) report a prominent alteration of resting-state functional connectivity between an amygdala seed region and posterior insular cortex, extending prior evidence from task-based activation studies in this patient group. Finally, Davey et al. (2012) provide evidence that adaptive changes in the functional connectivity of the subgenual cingulate cortex may be necessary to support intact executive function early in the course of major depressive disorder (MDD), prior to the emergence of characteristic cognitive deficits.

In a second study of MDD, Almeida et al. (2011) use dynamic causal modeling (DCM) to characterize changes in the effective connectivity (i.e., causal functional interactions) of a circuit linking ventral frontal cortical regions and the amygdala during emotional processing. Their findings shed light on how sex differences in the dynamics of this network may relate to the clinical phenomenology of female compared to male MDD.

Graph analytic techniques are featured in the work of Xie and He (2011) and Collin et al. (2011). Xie and He provide a primer on the basic principles of graph theoretic analysis of neuroimaging data, and comprehensively review the application of these techniques to study disturbances of structural and functional brain network connectivity and topology in Alzheimer's disease. In a resting-state fMRI study, Collin and colleagues use graph analytic techniques to provide some of the first evidence that functional connectivity between cerebellar and cortical and subcortical regions is associated with genetic risk for schizophrenia.

Finally, Lungu and Stip (2012) present an interesting case study of a schizophrenia patient with partial agenesis of the corpus callosum. They report intact activation to perception of visual stimuli but abnormal neural activation during emotional processing in this patient. They interpret this finding as implicating callosal connectivity in patients’ emotional and psychotic disturbances, a conclusion consistent with morphometric studies of callosal abnormalities in schizophrenia (Walterfang et al., 2008).

Collectively, the research presented in this Special Topic provides the novice reader with exposure to the diverse array of methods available for interrogating brain network structure and function in health and disease, and offers the expert reader prime examples of cutting-edge applications of these methods to understand psychiatric disorders. The continued development of novel connectivity mapping techniques (Bassett et al., 2011; Fornito et al., 2011a; Friston et al., 2011; Sui et al., 2012; Zalesky et al., 2012a), improvements in available algorithms and network models (Friston et al., 2011; Rubinov and Sporns, 2011; Zalesky et al., 2012b), and the elucidation of genetic influences on brain connectivity (Esslinger et al., 2009; Fornito et al., 2011b), will be critical for advancing work in this field. These advances, coupled with the valuable data to emerge from initiatives such as the Human Connectome Project, will enable researchers to move beyond localized, segregationist pathophysiological models of mental illness to characterize the full complexity of distributed neural circuit disruptions that underlie these conditions.

## Footnotes

^1^http://www.liebertpub.com/brain

^2^http://www.brain-connectivity-workshop.org/

^3^http://www.humanconnectomeproject.org/

^4^http://www.humanconnectome.org/

^5^https://sites.google.com/a/brain-connectivity-toolbox.net/bct/

^6^https://sites.google.com/site/functionalconnectivitytoolbox/

^7^http://fcon_1000.projects.nitrc.org/
